# Supplementary material for: Spectrum and Clinical Interpretation of TTN Variants in Ecuadorian Patients with Heart Disease: Insights into VUS and Likely Pathogenic Variants
Source: Int J Mol Sci. 2025 Dec 10;26(24):11896. doi: 10.3390/ijms262411896 (PMC12732953; doi:10.3390/ijms262411896)
Supplement: Supplementary file 1 [file ijms-26-11896-s001.zip › ijms-3991344-supplementary.pdf]

Supplementary Table S1. Ancestry component of the cohort.

Sample ID	AFR Component	EUR Component	NAM Component
ADN01	0.058	0.416	0.525
ADN08	0.034	0.525	0.441
ADN013	0.158	0.347	0.495
ADN014	0.046	0.535	0.419
ADN023	0.030	0.063	0.907
ADN037	0.024	0.177	0.799
ADN050	0.183	0.227	0.590
ADN058	0.533	0.215	0.252
ADN093	0.049	0.378	0.573
ADN097	0.113	0.356	0.531
ADN099	0.020	0.078	0.903
ADN100	0.028	0.421	0.551
ADN101	0.039	0.276	0.685
ADN103	0.113	0.100	0.787
ADN106	0.063	0.566	0.371
ADN116	0.025	0.053	0.922
ADN117	0.014	0.052	0.933
ADN120	0.017	0.037	0.946
ADN130	0.021	0.647	0.331
ADN146	0.018	0.084	0.898
ADN150	0.035	0.563	0.401
